# Magnetic Resonance Imaging as the Primary Imaging Modality in Children Presenting with Inflammatory Nontraumatic Atlantoaxial Rotatory Subluxation

**DOI:** 10.3390/children8050329

**Published:** 2021-04-23

**Authors:** Katharina J. Wenger, Elke Hattingen, Luciana Porto

**Affiliations:** Institute of Neuroradiology, University Hospital Frankfurt, Goethe University, 60528 Frankfurt am Main, Germany; elke.hattingen@kgu.de (E.H.); luciana.porto@kgu.de (L.P.)

**Keywords:** grisel syndrome, nontraumatic atlantoaxial rotatory subluxation, torticollis, computed tomography, magnetic resonance imaging, imaging protocol

## Abstract

Inflammatory nontraumatic atlantoaxial rotatory subluxation (AAS) in children is an often-missed diagnosis, especially in the early stages of disease. Abscess formation and spinal cord compression are serious risks that call for immediate surgical attention. Neither radiographs nor non-enhanced computed tomography (CT) images sufficiently indicate inflammatory processes. Magnetic resonance imaging (MRI) allows a thorough evaluation of paraspinal soft tissues, joints, and ligaments. In addition, it can show evidence of vertebral distraction and spinal cord compression. After conducting a scoping review of the literature, along with scientific and practical considerations, we outlined a standardized pediatric MRI protocol for suspected inflammatory nontraumatic AAS. We recommend contrast-enhanced MRI as the primary diagnostic imaging modality in children with signs of torticollis in combination with nasopharyngeal inflammatory or ear nose and throat (ENT) surgical history.

## 1. Introduction

Non-traumatic rotatory atlantoaxial subluxation (AAS) was first described in 1830 by Sir Charles Bell. He reported AAS in a patient suffering from a deep syphilitic ulcer of the pharynx [1]. The condition was later named after the Frenchman Pierre Grisel (1869–1959) who reported two patients with AAS coinciding with nasopharyngeal inflammation [2]. Since then, acquired Grisel syndrome has been described after otorhinolaryngological surgical procedures such as tonsillectomy and adenoidectomy [2,3,4,5], after nasopharyngeal soft tissue inflammation, Kawasaki syndrome (cervical lymphadenopathy, necrotizing microvasculitis with fibrinoid necrosis of mucosa of mouth and throat), and retropharyngeal abscess secondary to pulmonary tuberculosis [6,7,8]. Grisel syndrome is primarily found in the pediatric population with 68% of cases occurring in patients under the age of 12 years [9] and a total of 90% under the age of 21 [10]. The disease’s apparent age-dependency led to the introduction of a two-hit hypothesis [11]. A pre-existing cervical ligamentous laxity and longer alar ligaments in the pediatric population resulting in an atlas-dens interval that ranges up to 4.5 mm (adults 2.5 to 3 mm) could serve as a predisposition (“first hit”) [12]. The “second hit” could be caused by immune cells and inflammatory mediators that develop and maintain inflammatory response in pharyngeal tissue and spread through pharyngovertebral veins. Pharyngovertebral veins link posterior nasopharyngeal veins to the periodontoid vascular plexus. The plexus serves as drainage for the posterosuperior pharyngeal region. The lack of lymph nodes in the plexus allows a spread of the inflammatory response from the upper pharyngeal region to the atlantoaxial area. Inflammatory mediators can cause synovial and vascular engorgement, periligamentous inflammation, and edema, all of which might increase laxity of the transverse and alar ligaments [13]. Under normal circumstances, the posterior fibers of the alar ligaments retain the odontoid process in contact with the atlas and prevent anterior dislocation of the atlas on the axis [14]. The hypothesis of a “second hit” has been morphologically supported by a case report by Park et al. [15], who described enhancement of tissues surrounding the cervical spine on Magnetic Resonance Imaging (MRI) at initial diagnosis and its resolution on follow-up MRI three weeks later. MRI findings corresponded with the clinical presentation and resolution of torticollis in the patient.

The underlying pathophysiological principles suggest that MRI with its high sensitivity for soft tissue alterations is suitable as a primary diagnostic imaging modality for evaluation in Grisel syndrome. Nevertheless, radiographs and three-dimensional computed tomography (CT) are still methods of choice in many hospitals. We hypothesize that a combination of patient history, clinical findings, and standardized MRI allows early diagnostic success in this often-missed diagnosis and prevents exposure of children to ionizing radiation.

## 2. Materials and Methods

### 2.1. MR Imaging

MRI with its high sensitivity for soft tissue alterations has become the preferred modality for evaluation of the spinal cord, ligaments, and paraspinal soft tissues [16]. It allows assessment of paranasal sinuses, mastoid air cells, nasopharyngeal and upper neck soft tissue, transverse and alar ligaments, joint capsules of the cervical spine, as well as the cervical spinal cord. Inflammatory lesions of the upper neck or even abscess formation, periligamentous inflammation and edema, synovial inflammation, and laxity of the transverse and alar ligaments may support clinically suspected diagnosis of Grisel syndrome and facilitate treatment decisions. In the case of abscess formation, size and extent of fluid collection can be assessed as a preoperative measure.

**Figure 1:**
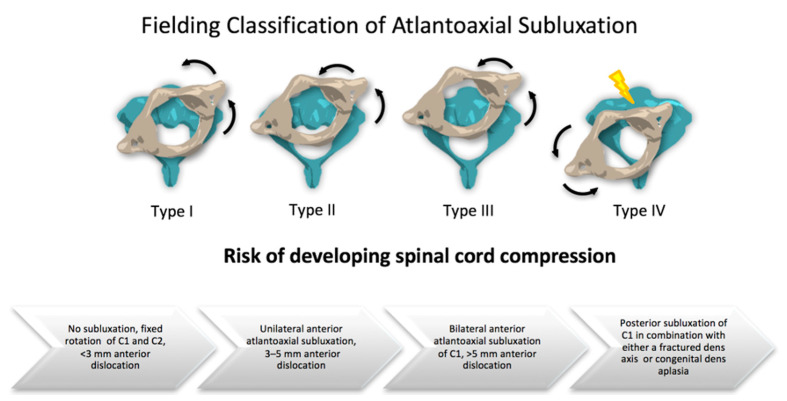
Fielding Classification of Atlantoaxial Subluxation (AAS) [17].

J. William Fielding, a clinical professor of orthopedic surgery, classified the rotatory subluxations of the atlantoaxial joint and described four lesion types that are shown in Figure 1. In Fielding Types III and IV, spinal cord compression may be present on MRI. Since clinical evaluation of damage to the dorsal column-medial lemniscus pathway and the spinothalamic tract can be difficult in young children, imaging plays an important role in diagnostics. According to Fielding et al., neurological complications occur in approximately 15% of cases. They can range from radiculopathy to myelopathy and may even be fatal [17]. Spinal cord lesions require surgical treatment with decompression and arthrodesis.

Initial diagnosis can be complimented by follow-up MRI of inflammatory lesions and abscesses for evaluation of treatment effects. Interventional MRI can be performed in case a therapeutic orthopedic procedure is indicated [18].

### 2.2. MRI Protocol

We performed a search in PubMed seeking all reports published until June 1st 2020 and examined all articles indexed with the medical subject or expert key words “Grisel syndrome” and “MRI” with text availability, “full text”. We included studies that mentioned or displayed the MRI sequences used for clinical diagnosis. Articles published in non-English languages were excluded. Eighteen studies met the inclusion criteria. 10/18 studies acquired T2-weighted (T2w) images in the sagittal plane, 5/18 studies in the axial plane and 4/18 studies in the coronal plane, making T2w images the most commonly used sequences. Post-contrast T1-weighted (T1w) images were again most commonly acquired in the sagittal plane with 7/18 studies. 4/7 studies used fat saturation in post contrast imaging. Details on included reports are shown in Table S1 [5,7,15,16,19,20,21,22,23,24,25,26,27,28,29,30,31,32].

## 3. Results

Based on the synthesized findings of our topic-based scoping review, and along with scientific and practical considerations, we outlined a standardized MRI protocol (Table 1) for suspected inflammatory nontraumatic AAS. The protocol can be applied to children of all ages. It can be used to support initial diagnosis and for routine follow-up. As a trade-off between large tissue coverage and the small size of spinal structures, 2D sequences were recommended with a maximum slice thickness of 3 mm [33]. For smaller children, a slice thickness of 2 mm should be considered. Minimum recommended sequences include sagittal and axial 2D T2-weighted (T2w) turbo spin echo (TSE), coronal 2D Turbo inversion recovery magnitude (TIRM) with short inversion time for fat suppression of bone marrow, sagittal pre contrast agent (CA), as well as sagittal, axial and coronal post CA 2D T1-weighted (T1w) TSE sequences. Scan time at 1.5 T amounts to approximately 23 min.

In case of insufficient image quality of 2D T2w sequences, 3D sequences utilizing balanced steady-state free precession (b-SSFP) such as constructive interference in steady-state (CISS) and fast imaging employing steady-state acquisition with phase cycling (FIESTa–c) are recommended. These sequences offer the ability to image with submillimeter spatial resolution [34].

### Illustration of Three Pediatric Cases of MRI in Inflammatory Nontraumatic Atlantoaxial Rotatory Subluxation

**Figure 2:**
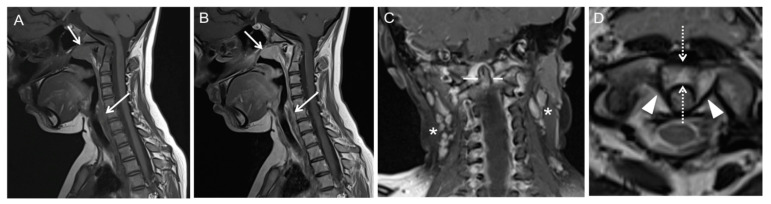
MRI shows edematous swelling and contrast enhancement of the posterior naso-, oro- and hypopharyngeal soft tissue (**A**,**B**); arrows; 2D T1w TSE pre and post CA. No abscess formation present. Enlarged cervical lymphnodes (**C**); asterisks; 2D T1w TSE post CA. Increased anterior atlantodental interval (**D**); dotted arrows; 2D T2w TSE. Asymmetry of odontoid lateral mass interval at midlateral mass level (**C**); line. Laxity of the transverse ligament of atlas (**D**); arrowheads. No spinal cord compression. T2w = T2-weighted; TSE = turbo spin echo; T1w = T1-weighted; CA = contrast agent.

Case 1: Patient history: Eleven-year-old patient with prior pharyngitis and cervical lymphadenopathy a month prior to acute torticollis. Three months had passed before the patient was referred to our hospital for further diagnosis. MRI findings are presented in Figure 2.

**Figure 3:**
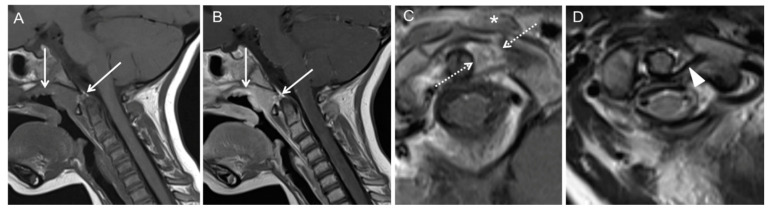
MRI shows edematous swelling and contrast enhancement of the posterior nasopharyngeal soft tissue with extension to anterior atlanto-occipital membrane and the cruciate ligament (**A**,**B**); arrows; 2D T1w TSE pre and post CA. Contrast enhancement of the left longus capitis (C); asterisk; 2D T1w TSE post CA signaling inflammation. No abscess formation present. Fixed rotation of C1 and C2 (**C**); dotted arrows; 2D T1w TSE post CA and synovial inflammation. Laxity of the transverse ligament of atlas (**D**); arrowhead; 2D T2w TSE. No spinal cord compression. T2w = T2-weighted; TSE = turbo spin echo; T1w = T1-weighted; CA = contrast agent.

Case 2: Patient history: Three-year-old patient with otolaryngological surgical procedure (nasal polypectomy) and postsurgical development of torticollis. Six weeks had passed before patient was referred to our hospital for further diagnosis. MRI findings are presented in Figure 3.

**Figure 4:**
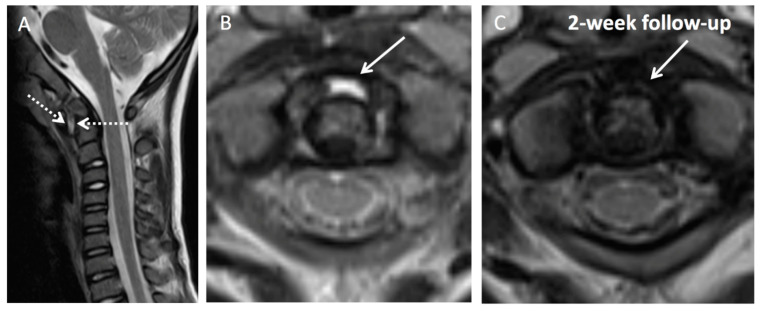
MRI shows edematous swelling of the posterior naso-, oro- and hypopharyngeal soft tissue with extension to the cruciate ligament (**A**); 2D T2w TSE. Joint effusion in the anterior synovial compartment of the dens seen as high T2 signal (**B**); arrow; 2D T2w TSE. No abscess formation present. Forward movement of C1 with increased anterior atlantodental interval (**A**); dotted arrows. No spinal cord compression; 2-week follow-up with appropriate empiric antibiotic regimen shows full regression of joint effusion (**C**); 2D T2w TSE. T2w = T2-weighted; TSE = turbo spin echo; T1w = T1-weighted; CA = contrast agent.

Case 3: Patient history: Six-year-old patient with prior pharyngitis and cervical lymphadenopathy prior to acute torticollis. Referral to our hospital after unsuccessful treatment with oral antibiotics from primary care physician. MRI findings are presented in Figure 4.

## 4. Discussion

The selection of an imaging modality in possible nontraumatic atlantoaxial rotatory subluxation is always preceded by clinical considerations [3,16,17,18]. Highly suggestive are a recent history of otorhinolaryngological surgical procedures (i.e., tonsillectomy and adenoidectomy) or an infection in the upper aerodigestive tract (i.e., tonsillitis). In addition, there should be typical clinical findings: a characteristic head position of 20° of tilt to one side, 20° of rotation to the opposite side, and slight flexion (torticollis). Laboratory findings are usually non-specific. Patients may show elevated C-reactive protein (CRP) levels and leucocyte counts in the first days of torticollis, followed by subsequent normalization of these parameters. Fever is rarely present.

Common imaging modalities include radiographs, CT, and MRI. The fixed posture in torticollis often causes technical difficulties obtaining correct radiographic projections in awake children. These technical limitations reduce their clinical importance compared to other modalities. In addition, radiographs are of minimal value in the first 4 weeks of disease, although flexion-extension views may be suggestive [35]. CT and MRI scans usually confirm the presence of rotational dislocation or anterior subluxation. Using CT, especially the dynamic solution with three scans in different head positions, exposes the often young patients to considerable levels of ionizing radiation [36,37]. It is therefore of high interest to reduce CT exposure settings in pediatric patients. Neither radiographs nor non-enhanced CT images sufficiently indicate inflammatory processes with possible abscess formation or myelopathy. MRI is well suited for the evaluation of nasopharyngeal regions of interest in Grisel syndrome. At the same time, MRI is adequate for visualizing atlantoaxial subluxation and spinal cord compression. Once diagnosis is established and in case a therapeutic orthopedic procedure is indicated, a reduction maneuver (maximal head rotation to the right first and then to the left during traction) can be performed immediately under general anesthesia with the patient lying in the scanner in a supine position. In this case, a single T2w sequence after the maneuver ensures a fast-operating time with reliable diagnostic accuracy [18].

Considering MRI as primary modality, it must be taken into account that especially smaller children can have a difficult time tolerating the unusual environment, loud noises, and the need to lie still [38]. In an emergency setting such as Grisel syndrome with acute inflammation, distraction and mock MR training techniques may not be applicable. It is therefore important to weigh pediatric sedation risks against radiation risks. In a meta-analysis conducted with data from the Pediatric Sedation Research Consortium of pediatric sedation/anesthesia encounters outside of the operating theater (>60% radiological procedures), serious adverse events were rare and no deaths occurred. Events that have the potential to harm and may require timely rescue interventions occurred once per 89 sedation encounters but could be well managed by a team of specialized care takers.

The standard diagnostic protocol we suggest can be acquired in approximately 20 min and weighs image quality/resulting diagnostic accuracy against scan time. Ligaments and symmetry of the odontoid lateral mass and anterior atlantodental interval are usually best evaluated on T2w sequences. Both fast spinecho inversion-recovery and T2w sequences are sensitive for bone marrow edema [39]. These advantages are reflected in the common use of both sequences in literature. T1w sequences without and with CA delineate the anatomy and pathologic conditions of the retropharyngeal and prevertebral spaces [40]. The protocol allows for image subtraction on the sagittal plane to identify contrast enhancing tissue and rim enhancement of abscess formation. Subtraction imaging is a technique whereby an unenhanced T1w sequence is digitally subtracted from the identical sequence performed after CA administration [41]. Post CA fat saturated T1w sequences were not included in the protocol, because the time required for application of the saturation pulse would substantially increase the imaging time [42]. However, as revealed by our literature review, some radiologists prefer the usage of fat saturated images for diagnostic work up. An alternative to 2D T1w sequences without and with contrast on sagittal plane are contrast-enhanced chemical shift techniques (Dixon) combining three echoes acquired at different echo times to create water-only and fat-only images [43].

## 5. Conclusions

We weighed pediatric sedation risks against radiation risks and MRI advantages over CT evaluating paraspinal soft tissues, joints, ligaments, and signs of spinal cord compression. In conclusion, we recommend contrast-enhanced MRI as the primary diagnostic modality in children with signs of torticollis in combination with nasopharyngeal inflammatory or ENT surgical history.

## Figures and Tables

**Table 1:** Standardized MRI (Diagnosis and Routine Follow-Up). Suggested standard protocol with a duration of approximately 23 min scan time at 1.5 Tesla (T). For smaller children, a slice thickness of 2 mm should be considered.

Sequence	Plane	Slice Thickness	Pixel Size	TR; TE; Number of Averages	Trans-mitting Coil	Receiving Coil	Comment
2D T2w TSE	sagittal	3 mm	1 mm	3800; 84; 2	body	neck	Aim to include nasopharyngeal and upper neck soft tissue, mastoid air cells and if possible paranasal sinus FOV
2D TIRM	coronal; planning on sagittal plane, block position angled parallel to cervical spine	3 mm	1 mm	3800; 33; 2	body	neck	Short inversion time (160 ms) for fat suppression of bone marrow
2D T1w TSE	sagittal	3 mm	1 mm	550; 9.5; 3	body	neck	
2D T2w TSE	axial; planning on sagittal plane, block position angled perpendicular to cervical spine	3 mm	1 mm	4500; 84; 2	body	neck	Include at least craniocervical transition to C3 and any pathological region on sagittal plane
2D T1w TSE post CA	sagittal	3 mm	1 mm	550; 9.5; 3	body	neck	
2D T1w TSE post CA	axial; planning on sagittal plane, block position angled perpendicular to cervical spine	3 mm	1 mm	550; 9.9; 2	body	neck	include at least craniocervical transition to C3 and any pathological region on sagittal plane
2D T1w TSE post CA	coronal; planning on sagittal plane, block position angled parallel to cervical spine	3 mm	1 mm	524; 17; 2	body	neck	

T2w = T2-weighted; TSE = turbo spin echo; TIRM = turbo inversion recovery magnitude; FOV = field of view; STIR = short-TI inversion recovery; T1w = T1-weighted; CA = contrast agent.

## Supplementary Materials

The following are available online at https://www.mdpi.com/article/10.3390/children8050329/s1, Supplementary Table S1: Details on included studies in scoping review of MRI in Grisel syndrome.

## References

[1] Dirheimer Y. (1977). The Craniovertebral Region in Chronic Inflammatory Rheumatic Diseases.

[2] Grisel P. (1930). Enucleation de l’atlas et Torticollis Nasopharyngien. PresseMed.

[3] Barcelos A.C.E.S., Patriota G.C., Netto A.U. (2014). Nontraumatic Atlantoaxial Rotatory Subluxation: Grisel Syndrome. Case Report and Literature Review. Glob. Spine J..

[4] Spennato P., Nicosia G., Rapanà A., Cicala D., Donnianni T., Scala S., Aliberti F., Cinalli G. (2015). Grisel Syndrome Following Adenoidectomy: Surgical Management in a Case with Delayed Diagnosis. World Neurosurg..

[5] Pavlidis E., Copioli C., Spagnoli C., Mazzotta S., Ormitti F., Crisi G., Pisani F. (2015). A Painful Stiff Neck Following an Ear, Nose, and Throat Surgical Procedure: Case Report. Neuropediatrics.

[6] Wood A.J., Singh-Grewal D., De S., Gunasekera H. (2013). Kawasaki Disease Complicated by Subluxation of Cervical Vertebrae (Grisel Syndrome). Med. J. Aust..

[7] Lee J.K., Oh C.H., Park H.-C., Yoon S.H. (2015). Grisel’s Syndrome Induced by Mycobacterium Tuberculosis. Korean J. Spine.

[8] Martins J., Almeida S., Nunes P., Prata F., Lobo M.L., Marques J.G. (2015). Grisel Syndrome, Acute Otitis Media, and Temporo-Mandibular Reactive Arthritis: A Rare Association. Int. J. Pediatr. Otorhinolaryngol..

[9] Gourin C.G., Kaper B., Abdu W.A., Donegan J.O. (2002). Nontraumatic Atlanto-Axial Subluxation after Retropharyngeal Cellulitis: Grisel’s Syndrome. Am. J. Otolaryngol..

[10] Wilson B.C., Jarvis B.L., Haydon R.C. (1987). Nontraumatic Subluxation of the Atlantoaxial Joint: Grisel’s Syndrome. Ann. Otol. Rhinol. Laryngol..

[11] Battiata A.P., Pazos G. (2004). Grisel’s Syndrome: The Two-Hit Hypothesis—A Case Report and Literature Review. Ear Nose Throat J..

[12] Kraft M., Tschopp K. (2001). Evaluation of Persistent Torticollis Following Adenoidectomy. J. Laryngol. Otol..

[13] Yu K.K., White D.R., Weissler M.C., Pillsbury H.C. (2003). Nontraumatic Atlantoaxial Subluxation (Grisel Syndrome): A Rare Complication of Otolaryngological Procedures. Laryngoscope.

[14] Gray H., Standring S., Ellis H., Berkovitz B.K.B. (2005). Gray’s Anatomy: The Anatomical Basis of Clinical Practice.

[15] Park S.-H., Park S.-H., Lee S.-H. (2013). Grisel Syndrome: Pathophysiological Evidence from Magnetic Resonance Imaging Findings. Ann. Rehabil. Med..

[16] Harth M., Mayer M., Marzi I., Vogl T.J. (2004). Lateral Torticollis on Plain Radiographs and MRI: Grisel Syndrome. Eur. Radiol..

[17] Fielding J.W., Hawkins R.J. (1977). Atlanto-Axial Rotatory Fixation. (Fixed Rotatory Subluxation of the Atlanto-Axial Joint). J. Bone Jt. Surg. Am..

[18] Hannonen J., Perhomaa M., Salokorpi N., Serlo W., Sequeiros R.B., Sinikumpu J. (2019). Interventional Magnetic Resonance Imaging as a Diagnostic and Therapeutic Method in Treating Acute Pediatric Atlantoaxial Rotatory Subluxation. Exp. Ther. Med..

[19] Ugur H.C., Cağlar S., Unlu A., Erdem A., Kanpolat Y. (2003). Infection-Related Atlantoaxial Subluxation in Two Adults: Grisel Syndrome or Not?. Acta Neurochir..

[20] Wurm G., Aichholzer M., Nussbaumer K. (2004). Acquired Torticollis Due to Grisel’s Syndrome: Case Report and Follow-up of Non-Traumatic Atlantoaxial Rotatory Subluxation. Neuropediatrics.

[21] Panopalis P., Christopoulos S., Churchill-Smith M., Chankowsky J., Ménard H.A. (2005). Grisel’s Syndrome: Non-Traumatic Subluxation of the Atlantoaxial Joint. J. Rheumatol..

[22] Yamazaki M., Someya Y., Aramomi M., Masaki Y., Okawa A., Koda M. (2008). Infection-Related Atlantoaxial Subluxation (Grisel Syndrome) in an Adult with Down Syndrome. Spine.

[23] Salpietro V., Polizzi A., Granata F., Briuglia S., Mankad K., Ruggieri M. (2012). Upper Respiratory Tract Infection and Torticollis in Children: Differential Diagnosis of Grisel’s Syndrome. Clin. Neuroradiol..

[24] Di Cola F., Cutilli T., Paulis D.D., Galzio R.J. (2013). Image-Guided Transoral Biopsy in a Boy with Grisel’s Syndrome. J. Clin. Neurosci..

[25] Reichman E.F., Shah J. (2015). Grisel Syndrome: An Unusual and Often Unrecognized Cause of Torticollis. Pediatr. Emerg. Care.

[26] Kourelis K., Haronis V., Konandreas I., Kontrafouri A., Asimakopoulos A. (2015). Atypical Post-Adenoidectomy Grisel’s Syndrome in Crouzon Child with Kyphotic Skull Base. Auris Nasus Larynx.

[27] Allegrini D., Autelitano A., Nocerino E., Fogagnolo P., De Cillà S., Rossetti L. (2016). Grisel’s Syndrome, a Rare Cause of Anomalous Head Posture in Children: A Case Report. BMC Ophthalmol..

[28] Ozalp H., Hamzaoglu V., Avci E., Karatas D., Ismi O., Talas D.U., Bagdatoglu C., Dagtekin A. (2019). Early Diagnosis of Grisel’s Syndrome in Children with Favorable Outcome. Childs Nerv. Syst..

[29] Fath L., Cebula H., Santin M.N., Coca A., Debry C., Proust F. (2018). The Grisel’s Syndrome: A Non-Traumatic Subluxation of the Atlantoaxial Joint. Neurochirurgie.

[30] Chua A.J.K., Tan B.W.S., Tan T.Y., Heah H.H.W. (2019). Grisel’s Syndrome in an Adult after Endoscopic Nasopharyngectomy. Laryngoscope Investig. Otolaryngol..

[31] Spinnato P., Aparisi Gomez M.P., Molinari M., Mercatelli D., Bazzocchi A. (2019). Torticollis After a Somersault: A Case of Grisel’s Syndrome. Indian J. Pediatr..

[32] Chryssikos T., Pratt N., Howie B., Mushlin H., Sansur C. (2020). Open Reduction and Decompression of Atlantoaxial Subluxation with Basilar Impression Due to Grisel Syndrome Using the Cervical Management Base Unit. World Neurosurg..

[33] Saunders D.E., Thompson C., Gunny R., Jones R., Cox T., Chong W.K. (2007). Magnetic Resonance Imaging Protocols for Paediatric Neuroradiology. Pediatr. Radiol..

[34] Li Z., Chen Y.A., Chow D., Talbott J., Glastonbury C., Shah V. (2019). Practical Applications of CISS MRI in Spine Imaging. Eur. J. Radiol. Open.

[35] Bissonnette B. (2019). Syndromes: Rapid Recognition and Perioperative Implications.

[36] Banerjee P., Thomas M. (2019). CT Scans to Exclude Spine Fractures in Children after Negative Radiographs May Lead to Increase in Future Cancer Risk. Eur. J. Orthop. Surg. Traumatol..

[37] Brenner D., Elliston C., Hall E., Berdon W. (2001). Estimated Risks of Radiation-Induced Fatal Cancer from Pediatric CT. AJR Am. J. Roentgenol..

[38] Dong S.-Z., Zhu M., Bulas D. (2019). Techniques for Minimizing Sedation in Pediatric MRI. J. Magn. Reson. Imaging.

[39] Benedetti P.F., Fahr L.M., Kuhns L.R., Hayman L.A. (2000). MR Imaging Findings in Spinal Ligamentous Injury. Am. J. Roentgenol..

[40] Debnam J.M., Guha-Thakurta N. (2012). Retropharyngeal and Prevertebral Spaces: Anatomic Imaging and Diagnosis. Otolaryngol. Clin. N. Am..

[41] Eid M., Abougabal A. (2014). Subtraction Images: A Really Helpful Tool in Non-Vascular MRI. Egypt. J. Radiol. Nucl. Med..

[42] Delfaut E.M., Beltran J., Johnson G., Rousseau J., Marchandise X., Cotten A. (1999). Fat Suppression in MR Imaging: Techniques and Pitfalls. RadioGraphics.

[43] Ma J. (2008). Dixon Techniques for Water and Fat Imaging. J. Magn. Reson. Imaging.

